# The precedence of syntax in the rapid emergence of human language in evolution as defined by the integration hypothesis

**DOI:** 10.3389/fpsyg.2015.00271

**Published:** 2015-03-18

**Authors:** Vitor A. Nóbrega, Shigeru Miyagawa

**Affiliations:** ^1^Department of Linguistics, University of São PauloSão Paulo, Brazil; ^2^Department of Linguistics and Philosophy, Massachusetts Institute of TechnologyCambridge, MA, USA; ^3^Center for Research and Development of Higher Education, University of TokyoTokyo, Japan

**Keywords:** biolinguistics, language evolution, linguistics, emergent view of language evolution, word formation, compounding

## Abstract

Our core hypothesis is that the emergence of human language arose very rapidly from the linking of two pre-adapted systems found elsewhere in the animal world—an expression system, found, for example, in birdsong, and a lexical system, suggestively found in non-human primate calls (Miyagawa et al., 2013, 2014). We challenge the view that language has undergone a series of gradual changes—or a single preliminary protolinguistic stage—before achieving its full character. We argue that a full-fledged combinatorial operation Merge triggered the *integration* of these two pre-adapted systems, giving rise to a fully developed language. This goes against the gradualist view that there existed a structureless, protolinguistic stage, in which a rudimentary proto-Merge operation generated internally flat words. It is argued that compounds in present-day language are a fossilized form of this prior stage, a point which we will question.

## Introduction

Human language presents two primary features that must be accounted for by any theory that attempts to deal with its emergence: its recent evolutionary development, arising within the past 100,000 years (Tattersall, 2009, 2012); and its autapomorphic character—it is a trait found only in humans, and not shared with other branches of the same monophyletic group (Tallerman and Gibson, 2012). One point of contention is whether it emerged in a gradual or rapid manner. We will challenge the first view, arguing that human language emerged rapidly from the linking of two pre-existing finite systems, a Type E system for *expressive*, found, for instance, in birdsong (Berwick et al., 2011), and a Type L system for *lexical*, found in monkey calls (Seyfarth et al., 1980; Arnold and Zuberbühler, 2006; Manser, 2013). This is the Integration Hypothesis of human language evolution (Miyagawa et al., 2013, 2014).

The emergent view adopted in this article is directly opposed to the gradualist view of language evolution (Bickerton, 1990, 1995, 1998, 2000, 2014; Pinker and Bloom, 1990; Newmeyer, 1991, 1998; Pinker, 1994; Jackendoff, 1999, 2002; Tallerman, 2007; Hurford, 2012). The core idea of the gradualist approach is that the earliest stages of human language are comprised of a structureless protolanguage, a system with no syntax. For some adherents of the gradualist approach (especially Jackendoff, 1999, 2002, 2009; Progovac, 2006, 2008, 2009, 2010, 2012; Progovac and Locke, 2009), human language has undergone a sequence of stages, initially a one-word stage, then a two-words stage—a proto-syntax—characterized by the combination of single words into compounds through a rudimentary recursive *n*-ary operation that generates flat structures (Progovac and Locke, 2009; Progovac, 2012)^1^. This reflects the Darwinian view that evolution proceeds in a succession of slow, small changes.

The notion of “word” is unclear in such an approach, since the so-called “word” varies sharply cross-linguistically (Dixon and Aikhenvald, 2003). We set aside the notion “word” and consider a more basic primitive that typically denotes some concept, namely, the root. A root is not the same as a word, since a word that appears in sentences is associated with additional elements such as category (e.g., noun, verb, or adjective) and inflectional information (e.g., number, tense, case, etc.). Based on the Integration Hypothesis, the root is the L-layer, and elements such as the categorial and inflectional features comprise the E-layer.

These two layers are linked by a rule called Merge, which takes two items and combines them into an unordered set (Chomsky, 1995). This structure-building rule is what gives rise to a single non-finite generative-engine capable of yielding any sort of linguistic object, from simple words to compounds and phrases—thus, “words” do not precede syntax in language evolution, but they are derived by such a system (contra Bickerton, 1990, 2014). A word is internally complex, often as complex as an entire phrase, hence, in line with Di Sciullo's (2013, 2014) claims, there is no reason to consider them as “linguistic fossils” of a previous stage of syntax. The Integration Hypothesis adopts the Darwinian view of exaptation: something shaped by natural selection is coopted for a new use—in fact two such systems were coopted and integrated to give rise to the unique function of language.

## The integration hypothesis and the emergent view of language evolution

Miyagawa et al. (2013, 2014) propose that human language is composed of two components, E for *expressive*, and L for *lexical*, each of which has an antecedent in other animal species. The E-type is similar to birdsong, which displays specific patterns without “words,” so that birdsong has syntax without meaning (Berwick et al., 2012)^2^. The lexical component is related to those systems that employ isolated uttered units that correlate with real-world references such as the alarm calls of Vervet monkeys (Seyfarth et al., 1980).

This correlation finds evidence in any simple word or sentence of human language, since every simple word or sentence is composed of two layers of meaning: a *lexical* structure that contains the lexical meaning, and an *expression* structure that is composed of function elements that give shape to the expression (Halle and Marantz, 1993; Marantz, 1997, for word formation; Chomsky, 1995; Miyagawa, 2010, for sentences). In word formation, the root is the lexical layer, and the categorial and grammatical information concatenated above a root is the expression layer^3^.


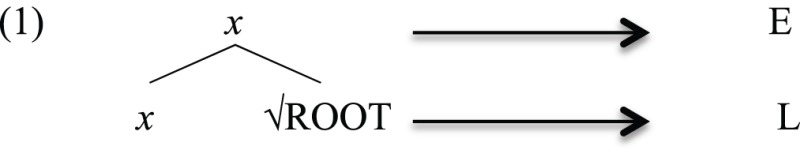


Since the root is assumed to lack syntactic category (i.e., nominal, verbal or adjectival; Marantz, 1997; Borer, 2003, 2005a,b, 2013), it must be conceived as a grammatical-free entity that itself does not show up directly in syntax^4^. Syntactic operations target “words” that have categorial and grammatical elements. The picture that emerges is the one in which the two systems, the L-Type and the E-Type, integrated to give rise to a single generative engine by way of the operation Merge. This integration occurred at the word level by combining roots with categorial and grammatical features.

The Integration Hypothesis is a more articulated version of the emergent approach (Berwick, 1998; Hauser et al., 2002; Chomsky, 2005, 2008, 2010, 2012, 2013; Berwick and Chomsky, 2011; Di Sciullo, 2011, 2013, 2014; Bolhuis et al., 2014). The emergent hypothesis assumes that the language faculty emerged late in historical development without any prior pre-syntactic stage. Its main operation is the recursive binary operation of Merge that derives hierarchical binary branching structures. This operation is triggered when one linguistic object β can satisfy a grammatical feature of a linguistic object α (Watanabe, 1996; Collins, 1997, 2002; Chomsky, 2000, 2004; Abels, 2003; Pesetsky and Torrego, 2006; Wurmbrand, 2014), as depicted in (2):


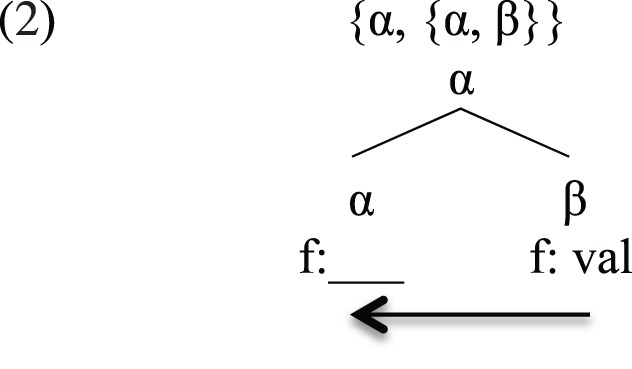


On this view, a root does not directly combine with another root in order to form a compound (Zhang, 2007), since a root is devoid of any grammatical feature. That is, the lexical primitives do not combine with each other to form purely lexical hierarchical structures, thus a sequence of L-layers is blocked in grammar. In fact, a typical sequence we find is E-L, where E furnishes the grammatical layer that allows elements to combine with each other (Miyagawa et al., 2013, 2014). The idea that two lexical primitives (i.e., roots) could combine would lead to unnecessary complexity: along with the conventional Merge that operates on grammatical (E) features, there needs to be a second type of Merge that combines roots. Also, there would be no way to distinguish different relations that exist between the members of a compound: predicate-argument, modification, coordination, etc. (Bisetto and Scalise, 2005; Guevara and Scalise, 2009)^5^.

The Integration Hypothesis characterizes the emergence of human language in one step. The pre-language stage is composed of root-like elements, each occurring in isolation of the others. It is possible that this is similar to the alarm calls, which apparently have reference in the real world, but are not associated with any grammatical features such as category. Thus, they do not participate in any combinatorial systems. Once the integration between the two systems, E and L, takes place, we have essentially all the features of a full-fledged human language. In its simplest form, this integration took place with the merger of a member of E and a member of L, forming the set {E, L}. At the word level, this means that roots combine with some grammatical features (GF).


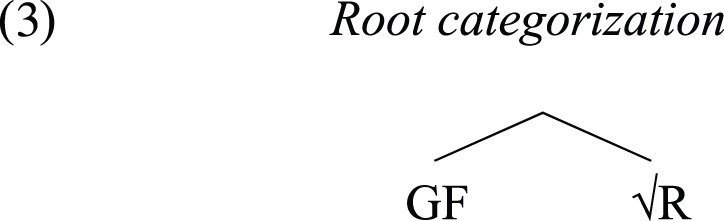


With a grammatical identity in place, the “word” is ready to participate in combinatory processes such as compounding and syntax. This can be illustrated by the overt realization of inflectional morphemes specific to certain syntactic categories in the compound's members. For instance, in the Turkish compound *gun-e bak-an* lit. day-DAT+look-PRT “sunflower” (Göksel, 2009), there is a dative case marker in the first member, and a participial suffix in the second member; the first indicates a nominal category and the second a verbal category, hence these are not roots.

Contrary to all these assumptions, Bickerton (1990, 1995, 1998, 2007, 2008, 2014) extensively argues that alarm calls hold distinct properties if compared to “words,” excluding any direct correlation between them, mainly because calls are genetically based^6^, while “words” are culturally based and also because calls are indexical, not symbolic. Assuming that the root is the closest approximation to calls, since they denote a conceptual content, we suggest that the linking of the L and E-Type systems might have enabled calls to expand its behavior due to the non-finite combinatorial system arising from the integration, which paved the way to the emergence of the open-vocabulary stored in our long-term memory, a point that still deserves more specific research^7^.

## Hierarchical structures within single words

Gradualist approaches rely primarily on the notion of “word” to determine the stages of human language evolution, beginning with the one-word stage, followed by a two-words stage made possible by concatenating two featureless “words” which we presume is close to today's roots. Nevertheless, it is difficult to assume that a primitive proto-Merge operation, characterized by generating flat structures, was at play in the concatenation of a root without grammatical information. Derived words, for example, show evidence for the presence of internal hierarchical structures within words, which must be constructed through a full-fledged operation Merge. An example is the presence of ambiguity in derived words, such as the adjective *unlockable*, which has different interpretations depending on the organization of its internal hierarchical structure. It can either mean that something cannot be locked, as in the configuration in (4a), or it may mean that something can be unlocked, as in (4b).


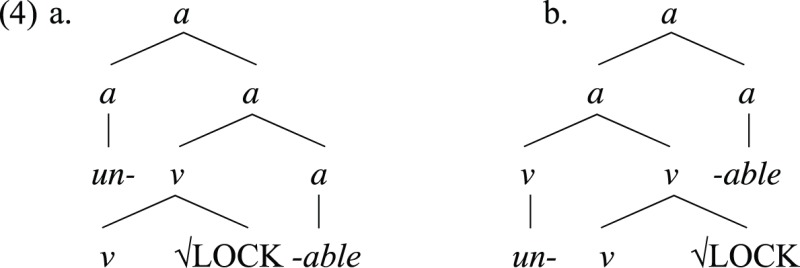


This ambiguity is due to the presence of a hierarchical structure within words, what would be impossible if words had a flat internal structure. Additionally, there is no reason a priori to assume that the merger of any categorial head in (4) can be different from each other (e.g., that there is a proto-Merge operation responsible for the first concatenation, but a full-fledged operation Merge responsible for the successive concatenations), since all category-defining heads refer to the same primitive. Besides, the assumption that both proto-Merge and Merge itself contribute to the derivation of linguistic objects, and that they coexistence as primitive operators of the same computational system, introduces complexity in the language faculty (Di Sciullo, 2013, 2014). In this sense, only a full-fledged operation Merge is directly responsible for the formation of words, always creating an endocentric hierarchical structure^8^.

## Against the view of compounds as “living fossils”

Compounds are frequently argued to be “living fossils” preserved from previous stages of a protolanguage (Jackendoff, 1999, 2002, 2009; Progovac, 2006, 2009, 2010, 2012; Progovac and Locke, 2009). These formations are considered to be the by-product of a raw concatenation, thus containing a flat internal structure derived by a proto-Merge operation. Jackendoff (1999, 2002, 2009), in particular, claims that compounds are a protogrammatical phenomenon, in which its rudimentary structure is not capable of shaping their semantic interpretation, so that the compounds' semantic interpretation is highly dependent on pragmatics. However, this dependence on pragmatics for meaning is not a universally attested property of compounds. While compound nouns from Germanic languages present a wide range of semantic interpretations (Downing, 1977; Allen, 1978), the same is not found in Romance languages compound nouns, which are very restricted in meaning (Bisetto, 2010; Delfitto et al., 2011). Thus, while the English compound *tree man* may have at least five possible semantic interpretations, for instance, (i) a man who is standing beside that tree, (ii) a man who is sitting on this tree, (iii) a man that usually seats on trees, (iv) a man who defends trees or forests and (v) a man resembling a tree, etc. (Delfitto et al., 2011), a Brazilian Portuguese compound such as *peixe-espada* lit. fish-sword “sword fish” can only mean “a fish resembling a sword.” This type of highly restrictive meaning associated with compounds is typical in Romance languages and casts doubt on the notion that compounds have no internal grammatical structure.

Progovac (2006, 2009, 2010, 2012) and Progovac and Locke (2009) argue that Slavic and English exocentric VN compounds (e.g., *daredevil, pickpocket*) are another example of relics from a protolanguage, because, for them, they have no internal hierarchical structure. The main arguments to assume that these compounds contain a flat structure are: (i) they are not recursive, (ii) they are no longer productive, and (iii) the thematic role of the noun is syntactically undetermined, which let it open to pragmatic interpretation^9^. With respect to (i), it is not true that exocentric VN compounds lack recursion at all^10^. We find recursion in these compounds in a variety of Romance languages, basically in two possible ways: (a) when a nominal exocentric VN compound becomes the complement of a verb, generating a new VN compound as in (5), and (b) when a noun internal to the compound contains a sequence of modifiers, as in (6a), or a list of complements, as in (6b). Recursion, and particularly the presence of self-embedding in such constructions, show that these compounds are derived by a full-fledged operation Merge, and must contain an internal hierarchical structure.


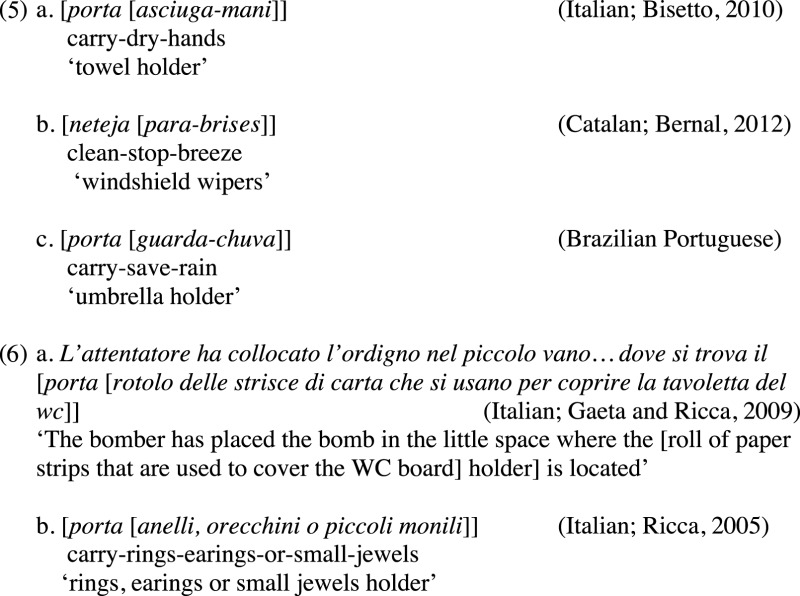


With respect to the lack of productivity of exocentric VN compounds, it is a language-specific distribution and it must be taken with caution. Although VN compounds are no longer productive in Slavic and Germanic languages, as well as in Chinese (Basciano et al., 2011), they are still very productive in Japanese, Romance^11^ and in some Bantu languages, such as Chichewa (Mchombo, 2004). In a wide typological research on the distribution of compounds cross-linguistically, Guevara and Scalise (2009) point out that the VN combination is the fourth most productive compound formation. In view of this, there is no reason to rely on a handful of languages in which these compounds happen to be no longer productive to argue that exocentric VN compounds lack internal structure. Cross-linguistically we find a rich word formation process that leads to the opposite conclusion.

Although VN compounds can be categorially, morphologically, and semantically exocentric, their internal structure is endocentric, since the verbal constituent maintains its head predicative character (Di Sciullo, 1991, 1992, 2005, 2013, 2014; Ferrari-Bridgers, 2005; Bok-Bennema and Kampers-Manhe, 2006; Gračanin-Yuksek, 2006; Nóbrega, 2014). The exocentric nature of VN compounds is due to the presence of an additional categorial layer attached above the VN combination, which provides a new categorial label to the endocentric VN structure, as we see in (7), and the insertion of inflection features in (8), following the general structure in (9).


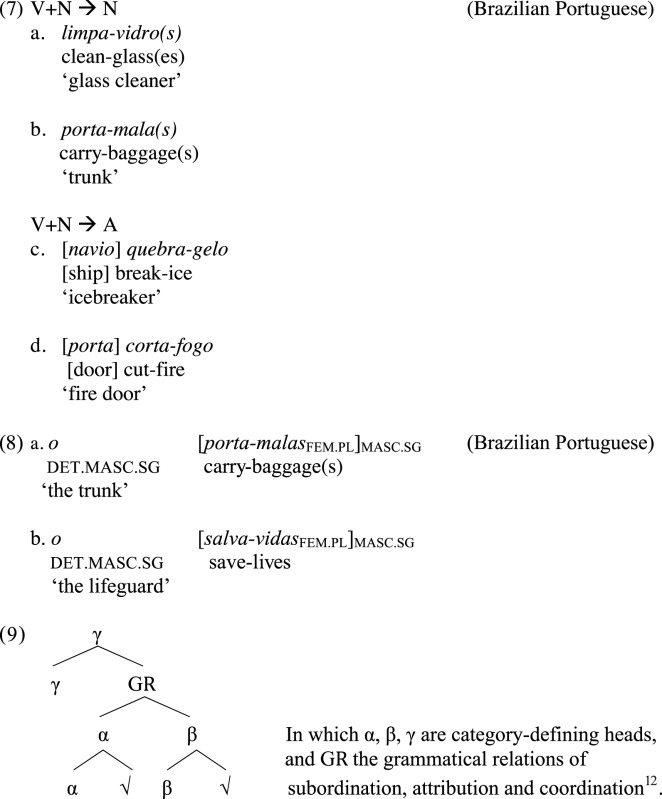


Additionally, if the noun internal to the compound can be interpreted as the agent of the verb, it is necessary to assume a hierarchical structure in order to differentiate these cases from those in which the noun is interpreted as the theme, as argued for by Di Sciullo (2013)^13^. Thus, in each case an unpronounced category is part of the compound structure, showing that differences in hierarchical structure are necessary for semantic interpretation^14^:


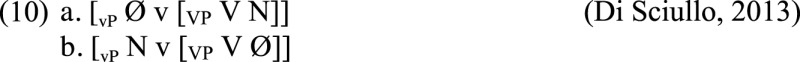


Besides, both arguments, internal and external, must be present within the compound. One piece of evidence is the impossibility of attaching an agentive suffix to these formations, which are considered to be the external argument in synthetic compounds (Di Sciullo, 1991, 1992). Thus, the ungrammatically of ^*^[[*lança-dor*]-*míssil*] lit. launch-missil “*missile launcher*” or of ^*^[[*míssil*]-*lançador*]] from the Brazilian Portuguese exocentric VN compound *lança-míssil*, is related to the fact that the external argument is being saturated twice, for a null pronoun *pro* and for the agentive suffix -*dor*^15^. These points invalidate the argument that these compounds are a sort of “living fossils,” since a cross-linguistic inspection shows that their properties are solely explained if an internally complex hierarchical structure exists.

## Conclusion

We challenged the view that compounds are “linguistic fossils” from the very beginning of syntax, in turn challenging the view that there existed protolinguistic stages in human language evolution. Internal complexity is found not only in compounds, but also in all their constituent members, a fact that weakens the assumption of a lexical protolanguage based on “words.” Since hierarchical structure can be attested in any single derived word, a full-fledged operation Merge can be assumed to be directly responsible for their formation, and consequently for the formation of compounds and phrases. This is the result of the integration between two pre-adapted systems, L and E, which allows the generation of all the linguistic objects present in modern human language.

### Conflict of interest statement

The authors declare that the research was conducted in the absence of any commercial or financial relationships that could be construed as a potential conflict of interest.

## Footnotes

^1^Jackendoff (1999, 2002), in particular, envisages not only two but at least nine partially ordered steps in human language evolution.

^2^Although parallels between birdsong and human language have often been suggested in the literature (Darwin, 1871; Jespersen, 1922; Marler, 1970; Nottebohm, 1975; Doupe and Kuhl, 1999; Okanoya, 2002; Bolhuis et al., 2010; Berwick et al., 2012), we endorse the idea that the actual link is between birdsong and the expression structure portion of human language, as already argued for by Miyagawa et al. (2013, 2014). Besides, a recent research suggests that the Integration Hypothesis is in fact on the right track. Using computational approach to gene expression profiles, Pfenning et al. (2014) have identified analogous brain regions for song and speech between birds and humans. This constitutes strong genetic evidence for half of the Integration Hypothesis, namely, the connection between birdsong and language.

^3^Mostly in accordance with a Distributed Morphology view of the primitives of the grammar (Halle and Marantz, 1993; Marantz, 1997; Embick and Noyer, 2007), and parallel with the Anti-Lexicalist Hypothesis put forth by Sigurðsson (2012).

^4^The acategorial nature of the root prevents it to contain grammatical and/or semantic operators, since this information would pre-specify its syntactic behavior and consequently would preclude its insertion in other categorial environments.

^5^The unnecessary complexity aforementioned comes from the assumption that bare roots could combine in a system where (i) roots are defined as a feature-less syntactic primitive, and where (ii) the combining operation requires feature valuation to apply (Chomsky, 2000; among others). Since roots are devoid of any grammatical feature, they cannot get into a feature valuation system, and consequently the merger of two bare roots is precluded by the syntactic component. If someone insists that the merger of two bare roots can be instantiated in the syntactic component, one needs to resort to the assumption of an additional underspecified Merge that blindly combines two feature-less syntactic objects, leading to such an unnecessary complexity. This additional operation is not limited to pair-Merge, and it must be powerful enough to distinguish the varied internal structural relations present within compounds, which would be made up of two bare roots, such as (a) the selection relation present in VN [e.g., (Brazilian Portuguese) *limpa-vidros* lit. clean-glasses “glass-cleaner”], NV [e.g., (Catalan) *cama-trencar* lit. leg(s)-break “break leg(s)”), and PN [e.g., (French) *contre-poison* lit. against poison “antidote”] compounds, and (b) the attributive, subordinate and coordinate relations within NN compounds (e.g., *sword fish, apron string* and *actor-director*, respectively), resembling grosso modo a proto-Merge operation.

^6^It is not consensual that call systems are entirely genetically based. Seyfard and Cheney (1986) suggest that the call system of Vervet monkeys may involve learning, using for evidence the fact that “when infants respond to the playback of an alarm call, they are significantly more likely to do so correctly if they have first looked at an adult.” Besides, “after an infant has given an eagle alarm call to a genuine predator, adults are significantly more likely to alarm-call themselves than when the infant alarm-call at a non-predator.” These facts suggest the presence of a mechanism of reinforcement in call systems, as pointed out by Schlenker et al. (2014).

^7^Recent works on the alarm-calling system of Campbell monkeys have analyzed alarm calls as roots or stems (Ouattara et al., 2009a,b,c; Schlenker et al., 2014). The main argument for such a treatment is the presence of affixation in such an alarm-calling system, in which the suffix -*oo* is argued to broaden the call's meaning. For instance, the call *krak*, used as leopard alarm, has its meaning broaden to a general call when affixed to -*oo*; and the call *hok*, an eagle alarm, becomes a general arboreal disturbance call after affixation. This particular distribution strengthens the assumption that alarm calls bear a parallel to roots, since they can be attached to recurrent dependent units.

^8^With respect to language ontogeny, the idea that a full-fledged Merge is the responsible for the derivation of words must also hold. Thus, the assumption that early child acquisition are a form of protolanguage (Bickerton, 1990, 1995) is wrong, since the child's ability of forming a simple word or of using inflected words presupposes the presence of a full-fledged structure-building operation able to combine a root with a category-defining head and subsequently with inflectional heads.

^9^The authors also argue that exocentric VN compounds from Slavic languages and English tend to display a derogatory semantics, being often coarse and vulgar when referring to humans. However, just a very few cases seem to display such interpretations in Romance agentive VN compounds. The many other interpretations that these compounds may have lack such a derogatory semantics, for instance, instrument, event, location, qualifying or relational adjective interpretations (Ricca, 2010). Further, the authors claim that the verb of Serbian VN compounds surface in the imperative form, which would evidence the fossilized nature of these compounds since the imperative mood is a protolinguistic form (Rolfe, 1996). However, such an analysis cannot be directly expanded to Romance exocentric VN compounds, since the verbal member of these compounds has no imperative semantic value (Scalise, 1992; Di Sciullo, 1992; Vogel, 1993; Vogel and Napoli, 1995; Peperkamp, 1997; Bisetto, 1999; Ferrari-Bridgers, 2005, for Italian; Fradin, 2009; Villoing, 2009, for French). Besides, in Italian, objects of imperative verbs cannot be bare nouns, contrary to the obligatory bare object condition of Italian VN compounds, for instance: *apri le bottiglie*! lit. open the bottles is a well-formed imperative sentence, while ^*^*appri bottiglie*! lit. open bottles is not, unlike its respective compound *apribottiglie* “can-opener” (Ferrari-Bridgers, 2005).

^10^Recursion is found in a variety of compounds, being very productive in some Germanic languages and in Turkish compound nouns (Don, 2009; Göksel, 2009; Neef, 2009; Bisetto, 2010).

^11^Except in Romanian, where VN compounds has a very limited productivity (Grossmann, 2012).

^13^Some VN compounds from Romance languages contain a nominal constituent interpreted as an adjunct (e.g., a locative, [French] *reveille* − *matin* lit. wake.up-morning ‘despertador’, Desmets and Villoing, 2009; [Chichewa] *chi*−*gonamb* á *wa* lit. sleep-bar ‘a drunk, an alcoholic’; Mchombo, 2004). We assume that these interpretations are the result of how the nominal constituent was merged to the verbal head, namely, either by set-Merge, when it is interpreted as the complement, or by a pair-Merge, when it is interpreted as an adjunct (Chomsky, 2000, 2004).

^14^Furthermore, the evidence that VN compounds are reduced relative clauses, due to their semantic exocentric interpretation (e.g., x such that [x [V N]]; Bok-Bennema and Kampers-Manhe, 2006), weakens drastically the idea that these compounds may have a flat internal structure, since the relative interpretation would never be derived in such flat structure.

^15^A null *pro* is also assumed to fill the external argument position of the verbal head in VN compounds to account for the fact that these compounds involve an agent and an instrument role. For instance, a [Brazilian Portuguese] *salva – vidas* lit. save-lives ‘lifeguard’ is someone who save lives, and a [Brazilian Portuguese] *limpa – vidros* lit. clean-glasses ‘glass cleaner’ is a product that clean glasses. Thus, the agent/instrumental role is structurally assigned to the specifier position of small *v*P. This implies that an empty DP that can bear this theta role must fill this position (Bok-Bennema and Kampers-Manhe, 2006), endorsing the presence of a more complex structure within these formations.
